# Internal Sulphate Attack in Masonry Mortars with Thaumasite Formation

**DOI:** 10.3390/ma15165708

**Published:** 2022-08-18

**Authors:** Servando Chinchón-Payá, Antonio Aguado de Cea, José Miguel Saval Pérez, José Servando Chinchón Yepes

**Affiliations:** 1 Eduardo Torroja Institute for Construction Sciences (IETcc-CSIC), Calle de Serrano Galvache, 4, 28033 Madrid, Spain; 2 Department of Environmental and Civil Engineering, Barcelona School of Civil Engineering (ETSECCPB-UPC), Jordi Girona 1–3, 08034 Barcelona, Spain; 3 Department of Civil Engineering, University of Alicante Polytechnic School, Carretera de San Vicente del Raspeig, s/n, San Vicente del Raspeig, 03690 Alicante, Spain; 4 University Institute of the Water and the Environmental Sciences of University of Alicante, Carretera de San Vicente del Raspeig, s/n, San Vicente del Raspeig, 03690 Alicante, Spain

**Keywords:** thaumasite, mortar, ettringite, solid solution, internal sulphate attack

## Abstract

The present paper focuses on the study of mortar samples where expansions with thaumasite formation occur as a consequence of sulphate attack. The samples correspond to a masonry mortar used in a rural construction located in the Spanish province of Toledo made of cement with limestone filler addition CEM II/AL. Composition and microstructure of the mortars have been analysed by means of scanning electron microscopy (SEM) using secondary and backscattered electrons (BSE) and X-ray diffraction (XRD). The results show that aggregates are contaminated with gypsum, which is the source of the sulphates for the internal attack. It seems that thaumasite is formed through an ettringite transformation where aluminium atoms are replaced with silicon atoms by means of a solid solution. The study highlights that thaumasite can be formed in warm weather through an internal sulphate attack due to gypsum contamination of aggregates.

## 1. Introduction

Thaumasite is a calcium carbonate silicate sulphate (Ca_3_Si(OH)_6_(SO_4_)(CO_3_)·12H_2_O) that appears in mortars and concretes due to sulphate attack under certain conditions. While ettringite formation has been widely studied—leading to a general consensus on the fact that it results from a reaction of sulphates with the hydrated calcium aluminate present in cements [1]—some controversy still exists with regard to thaumasite formation and its possible connection to ettringite. The current state of knowledge broadly revolves around these three hypotheses [2]: (a) through direct precipitation from its components in the cement matrix pore solution; (b) through an ettringite transformation or conversion in which aluminium atoms are replaced with silicon atoms forming an ettringite-thaumasite solid solution; (c) through ettringite too, but, unlike the previous case, the ettringite crystals now act as a nucleating medium from which thaumasite crystals grow. Laboratory studies have shown that thaumasite can form from its components in solution [3,4] but that it does so more rapidly or to a greater extent when ettringite is present in the reaction medium [5]. Unfortunately, case studies of thaumasite formation in real samples do not allow us to be more concise on the formation mechanism.

Numerous studies show that thaumasite is found in a variety of building materials ranging from gypsum plasters to structural concrete in contact with groundwater sulphates [6,7,8,9,10,11]. It has also appeared in historic buildings [12], especially in the presence of new conservation materials [13,14]. In relation to thaumasite components, hydrated calcium silicates C-S-H are the source of silicon used to form thaumasite. This is the main reason why thaumasite turns out to be so damaging. When C-S-H disappears, the cement loses its cohesive power, and concrete or mortar becomes a weak paste, which can be easily smashed with hand pressure.

The source of carbonate needed to form thaumasite may come from within the concrete itself in the form of aggregates or limestone fillers [15,16] or from external sources through atmospheric interaction [17] or the contact with CO_2_ dissolved in the groundwater for a long time [18].

The sulphates required to form thaumasite inside a structure might come dissolved in the water that is in contact with or has been incorporated into mortar or concrete during the manufacturing stage and, therefore, found inside the matrix as impurities. This can be due to an excessive utilization of gypsum as a retardant in cement manufacture or to the use of aggregates contaminated with gypsum or easily oxidisable iron sulphides.

The vast majority of studies and case reports of thaumasite formation have sulphates coming from an external source [19,20,21]. There are very few studies in the literature in which the cause of the appearance of thaumasite is due to internal sulphate attack. In a recent paper [22], the authors show an EDX image where they assume to have ettringite plus thaumasite, which appeared in mortars where recycled aggregates contaminated with gypsum were used.

Most of the studies conclude that thaumasite formation occurs to a greater extent at low temperatures (about 5 °C) due to its faster kinetics [9,23,24,25,26]. However, some cases of thaumasite appearance in the concrete of not very cold climates have been found, and some authors question whether low temperatures are a fundamental requirement [27].

The authors of this paper have extensive experience in the analysis of mortar and concrete pathologies. In particular, the authors have seen numerous cases of sulphate attack, some of them due to an internal source [28,29,30], such as when aggregates contain oxidisable iron sulphides [31,32,33,34,35,36].

The present work shows the study of a bonding mortar used for roof tiles and pavement in a building. The composition and microstructure of the mortar are analysed to determine the causes of deterioration. The results show that the main cause is the formation of thaumasite due to an internal sulphate attack by the use of aggregates contaminated with gypsum. The study also shows that low temperatures are not necessary to form thaumasite.

## 2. Materials and Methods

### 2.1. Samples

**Figure 1:**
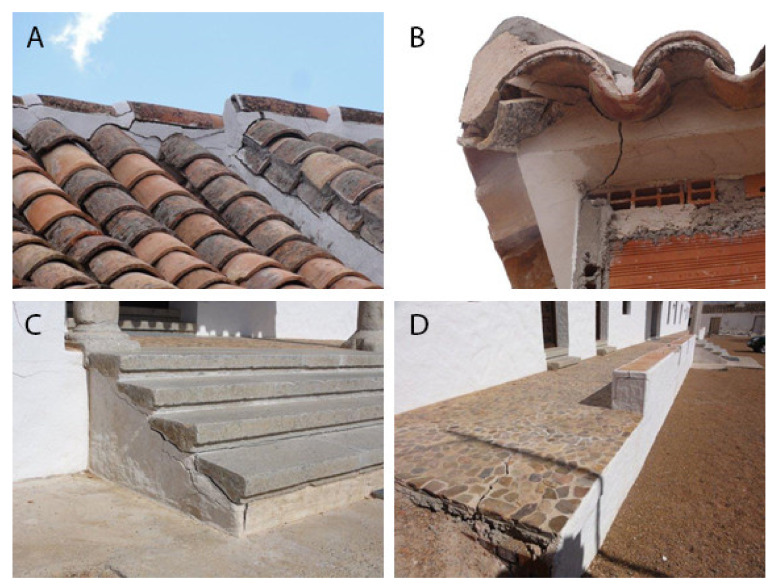
Photos showing the expansive nature of the mortar. (**A**) and (**B**) roof tiles, (**C**) and (**D**) paving tiles.

The property under study is called Quinto de Garcillán and is located in Toledo (Spain). It is an estate that houses private and public industries and is a typical example of rural architecture. The samples analysed in this study are of a masonry mortar used to place tiles and for pavements. The photographs in Figure 1. show the state of the mortar, with clearly visible expansions and cracks in many parts of the dwelling such as roofs (Figure 1A,B)) or pavements (Figure 1C,D)).

Samples of degraded mortar have been taken from the areas shown in Figure 1, as well as mortar from other apparently healthy areas to check the cause of the observed pathology.

**Figure 2:**
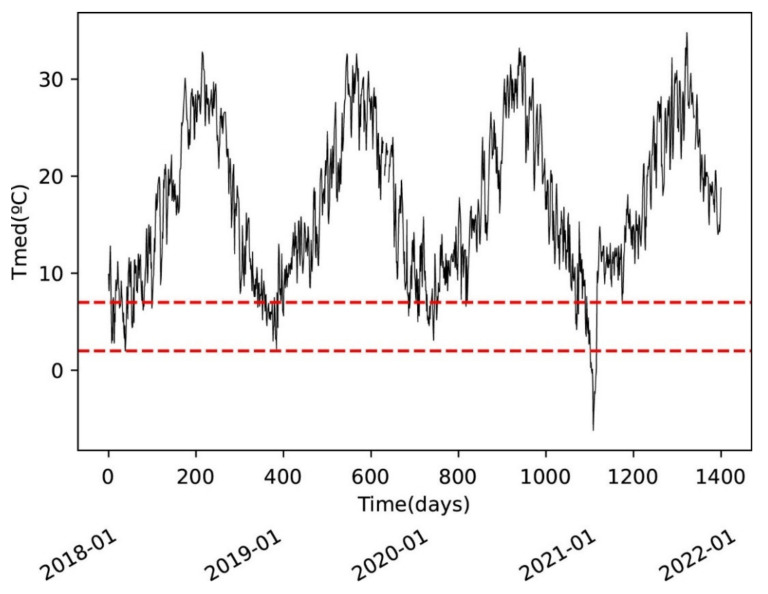
Daily average temperatures in the site.

Figure 2 shows the daily average temperatures of the location of the building. This area has temperatures ranging from 2 °C to 30 °C. The graph shows the lower range of temperatures, where thaumasite is told to be formed according to the vast majority of papers published.

As can be seen in Figure 2, very few days per year have temperatures of 5 °C, so it can be concluded that the area has warm temperatures in the context of thaumasite formation.

### 2.2. Experimental

- The in situ visual inspection of the property allowed the most affected areas to be identified. As has been pointed out, these correspond to the mortar of the roof tiles and paving, from which samples were taken for subsequent analysis in the laboratories and scientific–technical services of the University of Alicante. The analyses conducted were:
- Firstly, the samples have been visualised under an optical microscope to select the areas to be analysed later by Scanning Electron Microscopy (SEM-EDX) and X-Ray Diffraction (XRD), both in the Research Support Services of the University of Alicante (Spain).
- SEM-EDX analyses were carried out with a scanning electron microscope Hitachi S3000N model. This microscope has an X-ray detector Bruker brand XFlash 3001 model for microanalysis and mapping. The use of energy-dispersive X-rays (EDX) allowed us to obtain the images on which mappings of elements were subsequently superimposed. Both secondary electron analysis (SEM) and backscattered electron analysis (BSE) have been used.
- XRD analyses were performed on a Bruker D8-Advance with an X-ray generator KRISTALLOFLEX K 760-80F and a Cu Kα tube. The samples were crushed in an agate mortar until they reached a size of ca. 40 μm, after which a sweep was performed from 4 to 60 degrees of 2θ at a rate of 1 °/min.

The refinement of cell parameters in XRD spectrum was possible thanks to the XPowder12 software (by J Daniel Martín. Ronda 101, Atalaya 1 - 2ºA. 18003, Granada (Spain)) [37], which permits us to distinguish between pure thaumasite and a solid thaumasite-ettringite solution. Data from *Thaumasite* (PDF#4-13-2568) were used for cell parameter refinement in the thaumasite [38].

## 3. Results

### 3.1. Optical Microscopy (Stereomicroscope)

**Figure 3:**
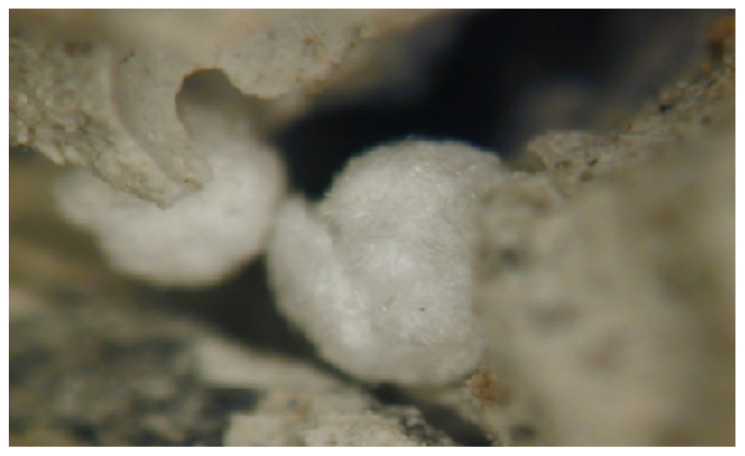
Acicular and white appearance of compounds observed by optical microscopy.

Detailed observation of the samples by stereomicroscope allows the identification of reaction products with a whitish and acicular appearance. These products appear abundantly in different areas of the surface observed in the samples (Figure 3). From the appearance indicated, these products could correspond to secondary ettringite and/or thaumasite crystals, both reaction products of a sulphate attack on the hydrated cement compounds.

### 3.2. Scanning Electron Microscopy (SEM and BSE)

**Figure 4:**
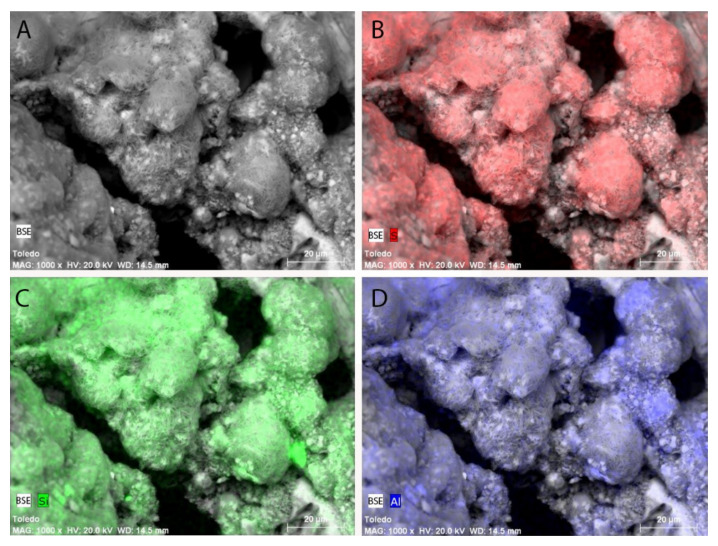
(**A**) Back-scattering electron image and mapping of the elements (**B**) sulphur, (**C**) silicon, and (**D**) aluminium.

Figure 4 provides the SEM-BSE image at 1000X of the altered mortar. It shows the mapping or element distribution in the surface by colouring sulphur, silicon and aluminium location.

**Figure 5:**
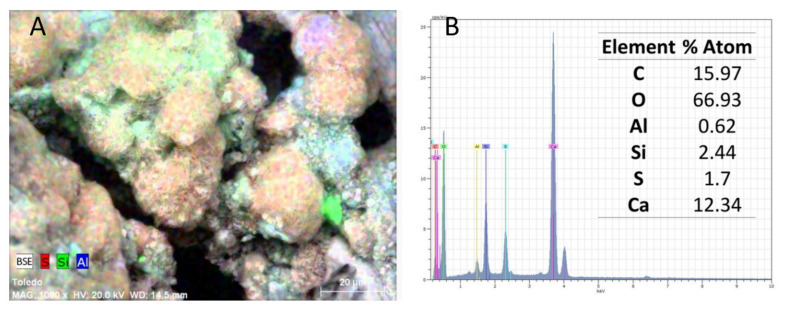
Combined mapping for elements S, Si, and Al; (**B**) EDX results for the area shown in (**A**).

Figure 5 describes the combined distribution of sulphur, silicon, and aluminium overlaid in a backscattered electron image and elemental composition obtained by EDX.

**Figure 6:**
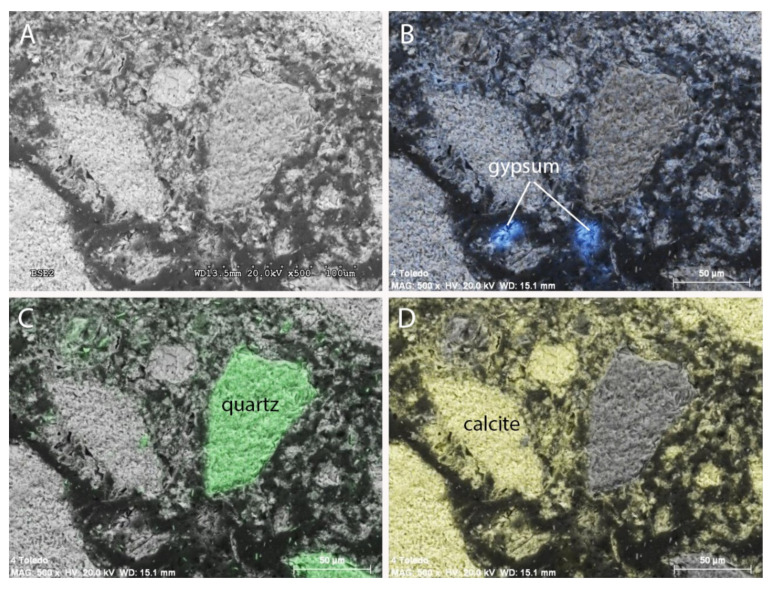
(**A**) Back-scattering electron image and mapping of the elements (**B**) sulphur, (**C**) silicon, and (**D**) calcium in the non-degraded mortar sample.

Finally, aggregate composition could be studied after using a brush and cleaning the altered surface in order to obtain a healthy portion of the mortar. BSE-SEM was used for the analysis. Figure 6 shows a BSE image with the Ca, S, and Si mappings superimposed on it.

### 3.3. X-ray Diffraction (XRD)

**Figure 7:**
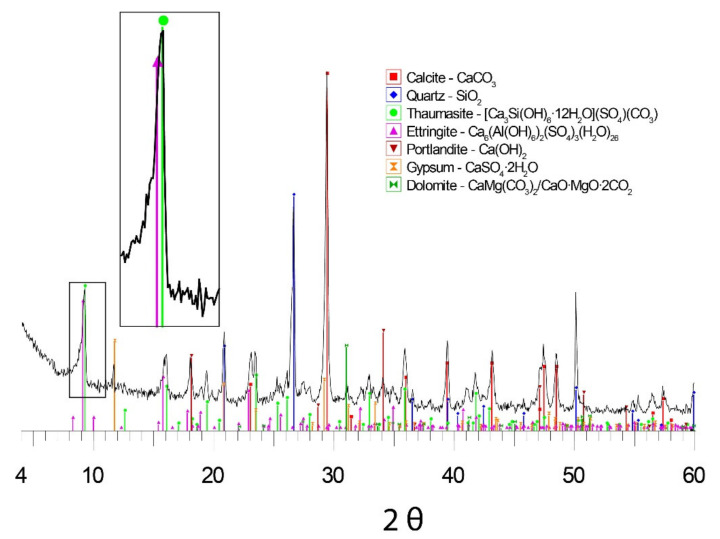
XRD spectrum of the cleaned mortar sample.

Figure 7 is one of the representative XRD results of a degraded mortar.

**Figure 8:**
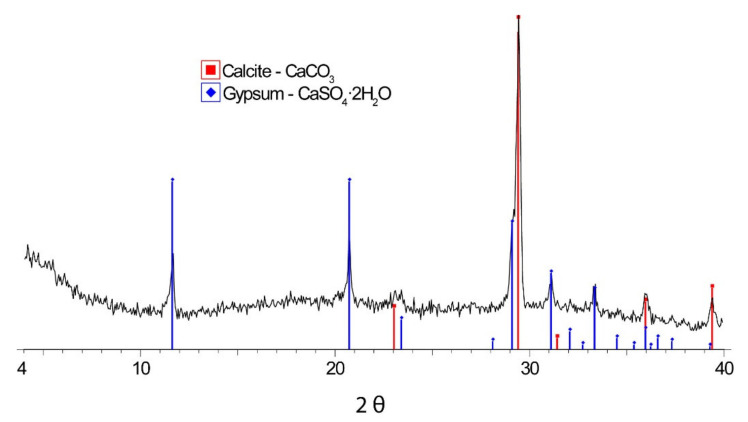
XRD of aggregates.

Figure 8 corresponds to the XRD analysis of the aggregates used to make the bonding mortars.

## 4. Discussion

The Al and Si mappings performed by BSE-SEM and represented in Figure 4. show that these elements occupy the same places. In principle, this would support one of the three hypotheses about formation given in the introductory section (mechanism b): Thaumasite is formed through an ettringite transformation or conversion where aluminium atoms are replaced with silicon atoms using an ettringite–thaumasite solid solution.

Figure 7 provides the XRD spectrum of a mortar obtained by quartering different mortar portions in the work site. Quartz, calcite, gypsum, ettringite, thaumasite, and portlandite were identified. The amount of ettringite and thaumasite detected in the sample is quite significant and corroborates that the cause of degradation of the mortar used is sulphate attack. It deserves to be highlighted in this respect that the presence of portlandite indicates that the mortar is not fully carbonated and would therefore come from a not-too-old set in use.

As reflected in the spectrum, ettringite and thaumasite coexist in the analysed sample. This fact could indicate that thaumasite is formed independently of ettringite (mechanism a) or due to ettringite crystals as the initial nucleation point (mechanism c). However, more extensive studies in the XRD pattern have been conducted to obtain further insights.

The XRD pattern in Figure 7 served to refine the thaumasite parameters using the data in the Powder Diffraction File with the software described in the Experimental section. Table 1 offers a summary of the results.

The results of cell parameter refinement for the XRD spectrum in Figure 7 show parameter “a” values below 1.111 nm—which would correspond to thaumasite–ettringite solid solution compounds according to [39]. Furthermore, the EDX analyses collected in Figure 5 reveal a Si/Al proportion that equals 3.94—corresponding to the values of this parameter [40].

The morphology of the aggregates, with well-defined angles and straight sides, represented in the picture provided in Figure 6 fits in with the gravel type. The mappings in Figure 6 as well as the DRX spectrum in Figure 7 prove the variable mineralogy of such aggregates. Gypsum is present among those minerals.

The amount of gypsum in the analysed samples is significant and is the only possible source of the sulphates that caused the damage. It can then be considered that the source of sulphates is internal to the composition of the mortars, and therefore an internal sulphate attack with thaumasite formation has occurred.

## 5. Conclusions

This paper describes a case report of internal sulphate attack in a masonry mortar with thaumasite formation. The mortar has been used as a bonding mortar for external paving and roof tiles, and in the most affected areas, there is extensive cracking and weakness in the bond with consequent detachment.

Samples have been taken from different affected areas. Optical microscopy analyses show the formation of a large number of acicular-type products corresponding to the products of sulphate attack. The results of scanning electron microscopy and X-ray diffraction allow us to identify that the mortar was made using cement with addition of limestone filler and a gravel aggregate contaminated with gypsum. These analyses confirm the first impressions after in situ visual inspection and optical microscopy of a degradation by sulphate attack, after which a large amount of secondary ettringite and thaumasite appears.

The fact that the temperatures in the area where the building is located tend to be above 5 °C demonstrates that thaumasite con be formed in warm weather. Thaumasite formation results from an ettringite transformation where aluminium atoms are replaced with silicon atoms through a solid solution.

## Figures and Tables

**Table 1:** Cell parameter refinement of the thaumasite detected in the sample.

Thaumasite	a (nm)	b (nm)	c (nm)	α	β	γ	Vol (nm)^3^
Table Data	11.0575	11.0575	10.4163	90	90	120	1.10296
PDF 240038							
refinement	1.10430 ± 0.00210	1.10430 ± 0.00210	1.04138 ± 0.00204	90	90	120	1.09980

## Data Availability

The data presented in this study are available on request from the corresponding author.
